# A novel estimate of biological aging by multiple fitness tests is associated with risk scores for age-related diseases

**DOI:** 10.3389/fphys.2023.1164943

**Published:** 2023-05-09

**Authors:** A. Manca, G. Fiorito, M. Morrone, A. Boi, B. Mercante, G. Martinez, L. Ventura, A. P. Delitala, A. Cano, M. G. Catte, G. Solinas, F. Melis, F. Ginatempo, F. Deriu

**Affiliations:** ^1^ Department of Biomedical Sciences, University of Sassari, Sassari, Italy; ^2^ Department of Medicine, Surgery and Pharmacy, University of Sassari, Sassari, Italy; ^3^ Unit of Endocrinology, Nutritional and Metabolic Disorders, AOU Sassari, Sassari, Italy

**Keywords:** biological age-chronological age, aging, cardiovascular diseases, 6-min walking test, timed “up and go” test, ten meter walk test, handgrip test

## Abstract

**Introduction:** Recent research highlights the need for a correct instrument for monitoring the individual health status, especially in the elderly. Different definitions of biological aging have been proposed, with a consistent positive association of physical activity and physical fitness with decelerated aging trajectories. The six-minute walking test is considered the current gold standard for estimating the individual fitness status in the elderly.

**Methods:** In this study, we investigated the possibility of overcoming the main limitations of assessing fitness status based on a single measure. As a result, we developed a novel measure of fitness status based on multiple fitness tests. In 176 Sardinian individuals aged 51–80 years we collected the results of eight fitness tests to measure participants’ functional mobility, gait, aerobic condition, endurance, upper and lower limb strength, and static and dynamic balance. In addition, the participants’ state of health was estimated through validated risk scores for cardiovascular diseases, diabetes, mortality, and a comorbidity index.

**Results:** Six measures contributing to fitness age were extracted, with TUG showing the largest contribution (beta = 2.23 SDs), followed by handgrip strength (beta = −1.98 SDs) and 6MWT distance (beta = −1.11 SDs). Based on fitness age estimates, we developed a biological aging measure using an elastic net model regression as a linear combination of the results of the fitness tests described above. Our newly developed biomarker was significantly associated with risk scores for cardiovascular events (ACC-AHA: r = 0.61; *p* = 0.0006; MESA: r = 0.21; *p* = 0.002) and mortality (Levine mortality score: r = 0.90; *p* = 0.0002) and outperformed the previous definition of fitness status based on the six-minute walking test in predicting an individual health status.

**Discussion:** Our results indicate that a composite measure of biological age based on multiple fitness tests may be helpful for screening and monitoring strategies in clinical practice. However, additional studies are needed to test standardisation and to calibrate and validate the present results.

## Introduction

Persons of the same chronological age may vary in their pace of aging, suggesting that chronological age is an inadequate proxy of biological aging (Liu et al., 2018). Most researchers agree that biological or phenotypic aging can be described as the accumulation of damages at the cellular, molecular, tissue and organ levels, which lead to “age-related changes in an organism that adversely affect its vitality and functions” (Finch, 1994; Gilbert, 2000). The characteristics of biological aging, as distinguished from diseases of aging, affect all the individuals of a species.

The current gold standard for quantifying biological aging is DNA methylation (DNAm), which allows estimating chronological and biological ages through epigenetic clocks that can also predict a variety of aging outcomes, including all-cause mortality, cancers, health span, and physical functioning (Levine et al., 2018). Biological age can also be estimated via a newly introduced tool, the Phenotypic Age calculator, which considers a combination of blood-measured biomarkers associated with longevity (Levine et al., 2018). Only very recently, epigenetic clocks have started to incorporate physical fitness (PF) parameters into their calculations (e.g., the DNAm FitAge) (McGreevy et al., 2022). These measures have been shown to correlate with changes in molecular signs of decline and can provide further insights into the effect of lifestyle on the aging process.

The American College of Sports Medicine defines PF as “a set of attributes that people have or achieve that relates to the ability to perform physical activity. It is also characterized by 1) an ability to perform daily activities with vigor, and 2) a demonstration of traits and capacities that are associated with a low risk of premature development of hypokinetic diseases (e.g., those associated with physical inactivity)” (Wilder et al., 2006). Indeed, maintaining good levels of PF during adulthood and later life, i.e., active aging, helps preserve autonomy and functional abilities and decelerate aging trajectories (Pareja-Galeano et al., 2015; Fiuza-Luces et al., 2018). Adequate PF is considered an established protective factor against chronic diseases and age-related disabilities (Sanchez-Sanchez et al., 2020). Importantly, the amount of physical activity accomplished in the transition from adult to older age is crucial to fostering successful ageing and has been shown to surpass other cardiovascular or sociodemographic risk factors that are classically associated with adverse health outcomes (Sanchez-Sanchez et al., 2020). Health benefits of active aging include reduced hospitalization and mortality rates, increased lifespan, and quality of life (Ekelund et al., 2019; Sanchez-Sanchez et al., 2020). It has been pointed out that preventing loss of physical and cognitive function and improving mental health and social engagement are the main benefits whereby physical activity would mostly contribute to improved chances of successful and healthy aging (Szychowska and Drygas, 2022). Older adults who maintain a regular physically active lifestyle have been extensively reported to display estimated biological ages considerably younger than their chronological ages (Nakamura et al., 1989; Levine et al., 2018; Sanchez-Sanchez et al., 2020; World Health Organization, 2015). The World Health Organization (WHO, 2002) defines healthy aging, “as the process of developing and maintaining the functional ability that enables wellbeing in older age” (Beard et al., 2016). Within such multidimensional framework, where the aging person needs to stay active to remain a resource to families, communities and economies, PF has proved among the most important contributors to healthy aging (Tucker, 2017).

In light of the above background, defining PF levels accurately and reliably is, therefore, of critical importance. Even though PF can be determined by multiple components—the main ones being body composition, cardiorespiratory endurance, muscular strength, flexibility, and balance (Caspersen et al., 1985), the cardiorespiratory domain is by far the most examined. This is generally accomplished by means of the gold standard for cardiorespiratory fitness assessment, which is the maximum oxygen uptake obtained at the end of a cardiorespiratory exercise testing. In low-resource environments or the clinical setting, submaximal and field exercise tests are more feasible and generally preferred (Ross et al., 2016). Among these, the Six-Minute Walk test (6MWT) has emerged as the most employed test for cardiorespiratory fitness and overall functional capacity (Matos Casano and Anjum, 2022). The 6MWT was introduced by the American Thoracic Society in 2002 as a sub-maximal exercise test to assess aerobic capacity, endurance, and PF (ATS Committee on Proficiency Standards for Clinical Pulmonary Function Laboratories, 2002). Beyond assessing PF and the individual’s functional capacity, it also provides information regarding the systems involved in physical activity, including pulmonary and cardiovascular systems, blood circulation, body metabolism, and peripheral circulation (ATS Committee on Proficiency Standards for Clinical Pulmonary Function Laboratories, 2002). This has led to consider the 6MWT as a global mobility and PF test for both, healthy and diseased populations (Wiener et al., 2019; Soares-Miranda et al., 2021; Elshafey and Alsakhawi, 2022; Matos Casano and Anjum, 2022).

In the clinical setting, the 6MWT still provides the main definition of PF, despite this outcome is increasingly being assessed in research over different components (aerobic fitness, muscular strength and endurance, flexibility, and body composition). Some studies have recently attempted to overcome this limitation by employing more comprehensive testing procedures to determine PF in the elderly. Mack-Inocentio et al. (2020) evaluated PF in 590 older adults aged 60+ years through a multi-domain battery of as many as ten motor-functional tests and found that a smaller set of these (trunk strength, handgrip strength, 6MWT, sit-to-stand, sit-and-reach) could explain the largest amount of variation in physical performance and functional capacity of persons older than 60 years.

Based on the above background, we hypothesized that the new multi-domain definition of PF would outperform the mono-dimensional definition based on the 6MWT in predicting the abovementioned health risk scores of our mixed cohort of middle-aged and older adults. Therefore, the present study aimed at 1) profiling biological aging in a group in the 50–80 years through multiple fitness tests; 2) overcoming the main limits of the 6MWT and identifying a comprehensive and novel measure of biological aging based on different components of fitness; 3) identifying the best motor predictors of biological aging, and 4) testing their ability to estimate an individual state of health, though investigation of their association with cardiovascular, morbidity and mortality risk scores.

## Materials and methods

### Participants

**FIGURE 1:**
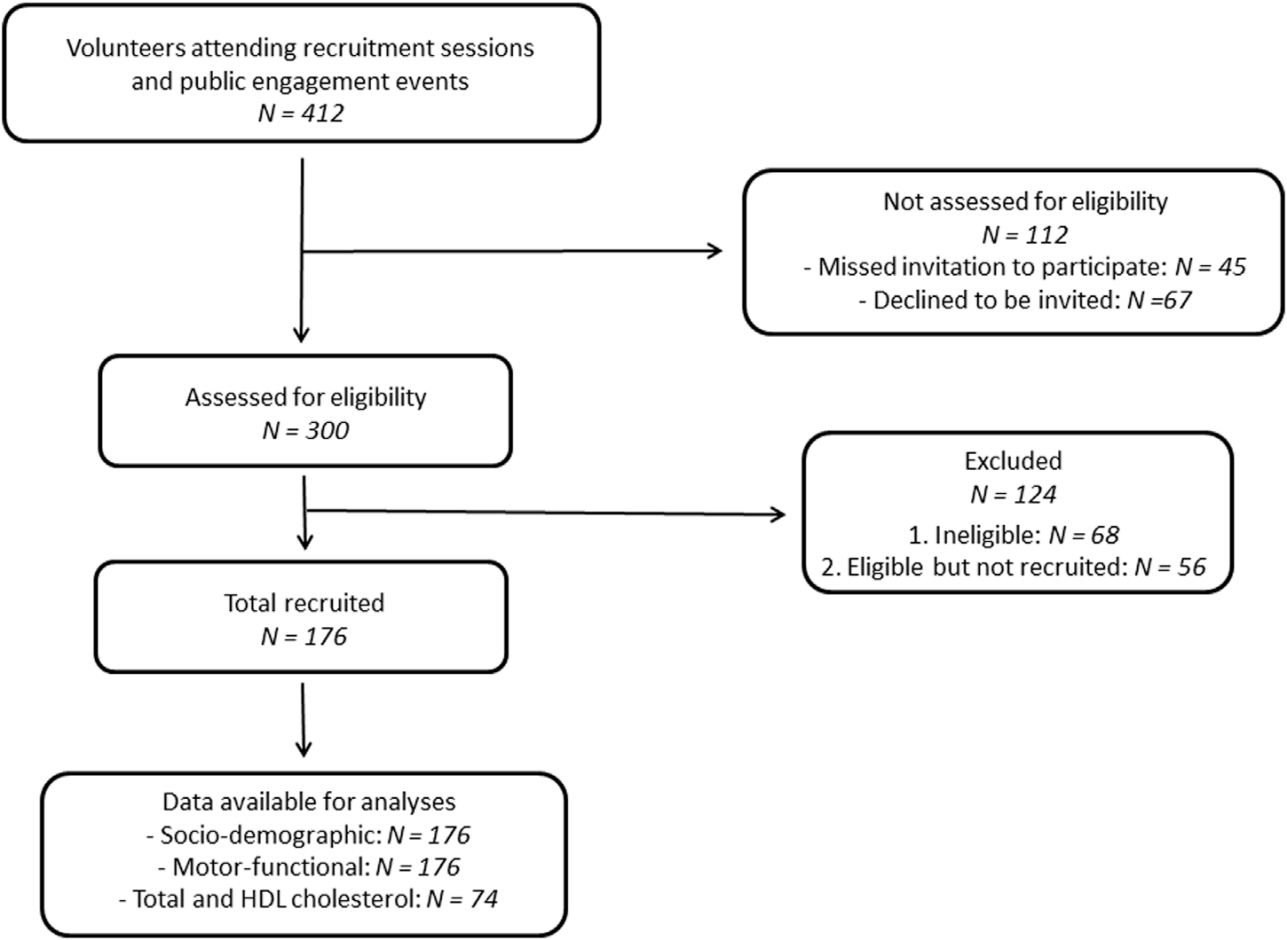
Study flow chart.

The current study was advertised via social networks and public engagement events to find participants. The group facilitator also gave a public lecture previewing the study project at the University of Sassari, Italy. Participants were required to be 50 years old or over at the time of the examination (from February 2021 to December 2021) and have no medical, physical, or cognitive condition that would interfere with participation in the functional assessments. We selected the first 300 respondents deemed apparently eligible after a preliminary telephone interview. After thoroughly screening for eligibility, 176 volunteers participated in the study. Figure 1 presents the study flow chart according to the Strengthening the Reporting of Observational Studies in Epidemiology (STROBE) checklist (Vandenbroucke et al., 2007). The Institutional Review Board and the Clinical Research Ethics Committee approved all procedures involving human subjects (ID: PG/2020/16846). Following the Declaration of Helsinki, written informed consent was obtained from each participant before inclusion and participation in the tests.

### Clinical examination

The subjects were evaluated by a geriatric specialist to ensure that they met the eligibility criteria. The patient’s heart rate and blood pressure were measured during the examination. At the same time, respiratory, rheumatologic, neurological, cardiovascular, musculoskeletal, neoplastic, and metabolic conditions were thoroughly investigated. Additional factors that may influence health outcomes, such as the participant’s smoking history, level of education, and current pharmacological therapy, were evaluated and recorded. The Italian validated version of the Geriatric Depression Scale was used to assess the subject’s depression (Galeoto et al., 2018). Lastly, adherence to the Mediterranean diet was evaluated using the MEDIET questionnaire, with scores ranging from low (0–5 points), medium (6–8 points), to maximal adherence (>9 points) (Ros et al., 2014).

### Motor-functional tests

Eight different tests were administered to examine the participant’s functional mobility, gait, aerobic condition, endurance, upper and lower limb strength and static and dynamic balance: 1) the Four Square Step Test (4SST) (Cleary and Skornyakov, 2017); 2) the Timed Up and Go test (TUG) (Podsiadlo and Richardson, 1991); the 10 m Walk Test (10MWT) evaluating both the 3) self-selected and comfortable walking speed and 4) fastest walking speed (Perera et al., 2006); 5) the Short Physical Performance Battery (SPPB) (Perera et al., 2006); 6) 6MWT (ATS Committee on Proficiency Standards for Clinical Pulmonary Function Laboratories, 2002); maximum voluntary isometric contraction of both the dominant 7) forearm (Handgrip test) and 8) quadriceps (Abizanda et al., 2012). Dynamometric measurements were performed with a Handgrip Dynamometer (G200, Biometrics LTD., Newport, United Kingdom) and with a hand-held dynamometer (MyoMeter M550, Biometrics LTD., Newport, United Kingdom) connected to a laptop via the same data collection tools.

Between each test repetition, a 1-min rest was given to recover, and a 2-min rest between one test and the next. The time taken to complete the tests was monitored using a stopwatch.

### Statistical analyses

#### Biological age definition

We defined the biological age of study participants based on the results of the motor-functional tests described above using a statistical approach previously employed to define the epigenetic age (Horvath, 2013). Specifically, we employed a linear regression model with elastic net regularization, where chronological age was the dependent variable (y), and the standardised results of the motor tests were the predictors (x_1_, x_2_, …). The elastic net model, including λ_1_ and λ_2_ penalisations, allows extracting relevant predictors of y and avoids overfitting simultaneously. The optimal values of the λ_1_ and λ_2_ parameters were derived as those minimising the root mean squared error (RMSQ) averaging from 100 permutations in which 80% of the sample was used (*glmnet R package*). The model-predicted age was defined as the biological/fitness age. By definition, individuals with higher predicted than chronological age are experiencing accelerated ageing and *vice versa*. Further, we determined the fitness age acceleration measure (fitAA) as the residuals of the regression of fitness age on chronological age.

#### State of health/risk scores

We computed three composite risk scores predictive of 10-year risk of cardiovascular diseases:

• the Framingham Risk Score (FRS) (D’Agostino et al., 2008),
• the Cardiovascular disease (CVD) risk from the American College of Cardiology (ACC) and American Heart Association (AHA) (Goff et al., 2013),
• the CHD risk prediction based on the MESA cohort (Budoff et al., 2018).

The three CVD scores include measures of total and HDL cholesterol, available for a subgroup of the whole study sample (N = 74). In this subsample, we computed the three CVD scores and a reduced version without using total and HDL cholesterol values. The CVD score calculated without cholesterol values had a Pearson correlation higher than 0.99 with the original measure for all three measures. Based on the above, we used the CVD risk score without cholesterol for subsequent analyses to increase the sample size. Also, we computed a composite score for the risk of diabetes within 7.5 years according to the algorithm described by Stern et al. (Stern et al., 2002). Similarly to what was described for CVD risk, the diabetes score version computed without cholesterol measures strongly correlated (R > 0.99) with the original one. Finally, we calculated the 10-year mortality score according to Levine et al. (Levine et al., 2018) and the Charlson Comorbidity Index (CCI) (Roffman et al., 2016).

## Results

This study sample included 176 volunteers (60,2% women) aged 51–80. Table 1 summarizes study sample characteristics, including anthropometric variables, health lifestyle variables such as smoking history, dietary status and polypharmacy and the maximum education attained as a proxy for the socio-economic status, and lifestyle. The average age was 66.5 years (SD = 7.8). Most of the study participants hold a high-school diploma (45%), whereas 5% attended primary school only; the average body mass index (BMI) was 26.9 kg/m^2^ (SD = 3.9); 56% were never smokers; finally, the average adherence to the Mediterranean diet score was moderate in women (median = 6, IQR = 2) and low in men (median = 5, IQR = 2).

**TABLE 1:** Anthropometric and demographic characteristics of the participants.

Variable (units of measurement)		Women N = 106	Men N = 70	Total N = 176
Age (years)		66.97 ± 7.46	65.74 ± 8.2	66.48 ± 7.77
		(65.53, 68.41)	(63.79, 67.7)	(65.33, 67.64)
Weight (kg)		63.55 ± 10.41	78.35 ± 12.22	69.44 ± 13.29
		(61.55, 65.56)	(75.43, 81.26)	(67.46, 71.41)
Height (m)		1.53 ± 0.09	1.69 ± 0.07	1.59 ± 0.11
		(1.51, 1.55)	(1.67, 1.71)	(1.58, 1.61)
Body mass index (kg/m^2^)		26.77 ± 4.17	27.27 ± 3.61	26.97 ± 3.95
		(25.97, 27.58)	(26.41, 28.13)	(26.38, 27.56)
Mediterranean diet adherence [pts: median (IQR)]		6 (2)	5 (2)	6 (2)
Comorbidity index (pts)		3.12 ± 1.68	2.71 ± 1.31	2.96 ± 1.55
		(2.8, 3.45)	(2.4, 3.03)	(2.73, 3.19)
Geriatric depression scale (pts)		4.65 ± 2.68	4.07 ± 2.72	4.42 ± 2.7
		(4.13, 5.17)	(3.42, 4.73)	(4.02, 4.83)
Polypharmacy (count)		2 (2)	1 (3)	1 (2)
Smoking history	Not smoking	*n* = 85	*n* = 62	*n* = 147
	Smoking	*n* = 18	*n* = 7	*n* = 25
	Never smoked	*n* = 57	*n* = 40	*n* = 97
	Have smoked	*n* = 46	*n* = 29	*n* = 75
Education level	Primary school	*n =* 6	*n =* 3	*n =* 9
	Middle school	*n =* 28	*n =* 12	*n =* 40
	High school	*n =* 44	*n =* 35	*n =* 79
	Tertiary +	*n =* 25	*n =* 17	*n =* 42

All data are presented as mean ± standard deviation and 95% confidence interval, except for polypharmacy data (expressed as median and IQR, interquartile range). Abbreviations: kg, kilograms; m, meters; pts, points.

### Fitness age definition and components

Our elastic net model extracted six features, as described in Table 2. A precise ranking was identified in how much each test contributed to fitness age. The weights in Table 2 can be interpreted as the increase in biological age for each increase by one standard deviation of the corresponding test result. Positive coefficients/weights indicate motor tests whose results are higher in an individual with higher fitness age and *vice versa*. Accordingly, TUG had the largest contribution, followed by handgrip strength and 6MWT distance.

**TABLE 2:** Coefficients of the elastic net model for selected variables contributing to the biological age (FitAge).

FitAge coefficients	
Variable	Coefficient (SDs)
TUG time	2.23
Handgrip strength	−1.98
6MWT distance	−1.11
Fast walking 10MWT time	0.95
4SST time	0.23
Quadriceps strength	−0.19

Abbreviations: SDs, standard deviations; TUG, timed up and go test; 6MWT, 6-min walk test; 10MWT, 10-m walk test; 4SST, four square step test. Coefficients indicate the standardized weight of each motor test in the construction of the biological/fitness age. Coefficients equal to zero indicate no contribution to the biological aging measure.

**FIGURE 2:**
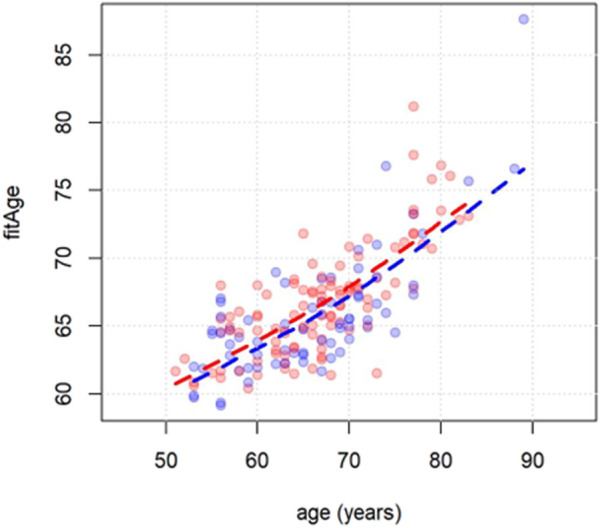
Scatterplot of chronological age (x-axis) vs. fitness age (y-axis). Men and women are indicated with blue and red dots respectively. Dashed lines are derived using the least squared error method.

As expected, the predicted (fitness) age correlated with the chronological age (Pearson 0.75, *p* < 0.0001; Figure 2). This applied both to women and men. Based on this relationship, the novel measure fitAA was derived as the residual of the linear regression of fitness age on chronological age.

### Association of fitAA with risk scores

In Table 3 we showed the results of the linear regressions of risk scores with fitAA adjusted for covariates as described in Methods. The results are presented in Table 3. Increased value of the fitAA was significantly associated with ACC-AHA and MESA scores for CVD risk, and with the Levine mortality score.

**TABLE 3:** Results of the linear models in which each risk score was used as the outcome (dependent variable) and fitness age acceleration (fitAA) as the predictor, adjusting for covariates.

	Estimate	95% CI	p
Framingham CVD score	0.26	(-0.04; 0.56)	0.0903
ACC AHA CVD score	**0.61**	**(0.27; 0.95)**	**0.0006**
MESA CVD score	**0.21**	**(0.08; 0.35)**	**0.0023**
Stern diabetes risk score	−0.25	(-1.14; 0.63)	0.5763
Comorbidity index	0.02	(-0.05; 0.08)	0.6469
Levine mortality score	**0.90**	**(0.43; 1.37)**	**0.0002**

Abbreviations: fitAA, fitness age acceleration; CI, confidence interval; CVD, cardiovascular diseases; ACC, American College of Cardiology; AHA, American heart association; MESA, multi-ethnic study of atherosclerosis. Estimates can be interpreted as the increase in the percentage risk score for each year increase in fitAA. Only significant p values should be in bold.

### FitAA vs. previous definition of physical fitness in predicting risk scores

Based on the results above, we propose to classify individual fitness status according to the newly developed fitAA measure. Specifically, we defined individuals with fitAA scores lower than −2.5 as “FIT”; individuals with fitAA scores ranging from −2.5 to 2.5 as “NORMAL”; and those with fitAA higher than 2.5 as “UNFIT”. Then, we compared the newly developed classification of fitness status with that commonly used in the literature based on the 6MWT (Matos Casano and Anjum, 2022) in the ability to predict risk scores for CVD, diabetes and mortality. Table 4 reports the results of the association of the categorical fitness status vs. risk scores, according to the two definitions described above.

**TABLE 4:** Results of the linear models in which each risk score was used as the outcome (dependent variable) and categorization of the fitness status as the predictor, adjusting for covariates. Estimates can be interpreted as the increase in the percentage risk score for individual in the NORMAL and UNFIT categories compared to the FIT (reference) group.

	Fitness status based on the 6 min walking test			Fitness status based on the fitAA measure		
	Estimate	95% CI	p	Estimate	95% CI	p
Framingham CVD score						
Normal	0.91	(−2.45; 4.28)	0.60	0.83	(−3.05; 4.71)	0.68
Unfit	−0.83	(−4.23; 2.57)	0.63	4.15	(−0.74; 9.04)	0.10
ACC AHA CVD score						
Normal	0.28	(−3.40; 3.95)	0.88	0.90	(−1.78; 3.57)	0.51
Unfit	−1.77	(−5.48; 1.94)	0.35	**4.51**	**(1.13; 7.89)**	**0.01**
MESA CVD score						
Normal	0.77	(−0.76; 2.30)	0.33	0.24	(−0.82; 1.31)	0.66
Unfit	0.42	(−1.13; 1.97)	0.60	**1.40**	**(0.05;2.74)**	**0.04**
Stern diabetes risk score						
Normal	−6.33	(−15.39; 2.72)	0.17	−1.99	(−8.87; 4.88)	0.57
Unfit	−6.52	(−15.68; 2.64)	0.17	−2.07	(−10.87; 6.72)	0.64
Comorbidity index						
Normal	0.13	(−0.48; 0.74)	0.67	0.16	(−0.36; 0.68)	0.55
Unfit	0.15	(−0.46; 0.77)	0.63	0.06	(−0.60; 0.71)	0.87
Levine Mortality score						
Normal	−0.34	(−4.82; 4.14)	0.88	0.27	(−3.50; 4.05)	0.89
Unfit	1.49	(−3.02; 6.01)	0.52	**4.80**	**(0.01; 9.60)**	**0.04**

Abbreviations: fitAA, fitness age acceleration; CVD, cardiovascular diseases; ACC, American College of Cardiology; AHA, American heart association; MESA, multi-ethnic study of atherosclerosis. Only significant p values should be in bold.

The fitness status definition based on fitAA outperformed the previous classification based on the results of the “6 min walking test” in estimating CVD and mortality scores, as shown in Table 4. In fact, no significant associations of risk scores with fitness status based on the 6MWT were detected, whereas fitness status based on fitAA was significantly associated with ACC-AHA and MESA CVD scores, and the Levine mortality score.

## Discussion

In this exploratory study we provide evidence that the individual biological age of our sample can be efficiently estimated by the comprehensive set of motor tests assessed here. The elastic net regression model identified the variables mostly contributing to biological age: the time needed to complete the TUG, and maximal handgrip strength, suggesting that in clinical practice, the results of these two tests deserve more attention for assessing an individual state of health. The strong association of these physical tests with biological age was not unexpected. Low grip strength at midlife may indicate subclinical disease, which later develops into clinical disease and disability, whereas good grip strength may mark some general intrinsic midlife vitality or motivation that tracks into good functional ability in old age (Rantanen et al., 1999). Handgrip strength, gait speed and ability to independently rise from a chair (the latter two being essential components of the TUG) are tests of muscle strength and function that have been recommended by international study groups on sarcopenia, including the European Working Group on Sarcopenia in Older People (EWGSOP) (Cruz-Jentoft et al., 2010), the Asian Working Group for Sarcopenia (Chen et al., 2014), and the International Working Group on Sarcopenia (Fielding et al., 2011) for the screening and diagnosis of sarcopenia. In this regard, handgrip strength is an established and powerful predictor of healthy aging beginning from midlife (Rantanen et al., 1999). Our findings on the relevance of TUG and handgrip also align with a more recent study where these tests were found to be the best predictors of short-term mortality in the elderly (Chua et al., 2020). They also agree with a pertinent consensus of experts, who proposed a panel of biomarkers of healthy ageing, which include the TUG and handgrip strength among the five biomarkers of physical capability (Lara et al., 2015). Interestingly, the other biomarkers are balance, gait speed and chair rising, all three being key components of the TUG.

Although preliminary, this finding suggests that these simple and well-known physical tests are strong and useful markers for predicting healthy aging trajectories. We also observed that the universally employed 6MWT was not a major contributor to age prediction. This disagrees with a relatively large and recent body of literature referring to this test as a measure of physical functioning and fitness (Wiener et al., 2019; Soares-Miranda et al., 2021; Elshafey and Alsakhawi, 2022). The gold-standard for demonstrating the physical fitness of an individual is the direct determination of peak oxygen uptake, which is considered the best indicator of cardiovascular fitness and aerobic endurance (Edvardsen et al., 2013). Due to the high costs and low feasibility of this approach in settings other than research, the walking distance covered in 6 min has been progressively supported as a low-cost, more applicable alternative to estimate fitness status, particularly in the elderly and diseased populations (Matos Casano and Anjum, 2022). However, age prediction models based on multiple domains are increasingly being favored over unidimensional measures as they can predict the individual health status in a more comprehensive manner. In this regard, Mack-Inocentio and colleagues introduced the Vitality Test Battery as a relatively simple tool that can be used to assess the physical condition of senior men and women outside a laboratory (Mack-Inocentio et al., 2020). Compared to their tool, which consists of a battery of 10 tests (6-min walk, trunk strength, hand grip strength, medicine ball throwing, 30-s chair stand, flexibility, balance, plate tapping, ruler drop, and dual task), our novel measure of fitness status based on the biological age, i.e., the fitness age acceleration (fitAA) that we developed, relies on two major contributors to fitness age, i.e., TUG test and handgrip strength, and to a minor extent, on the 6MWT. This measure was calculated into two steps: 1) an elastic net penalised regression model is trained to predict the chronological age of individuals; 2) the residuals of the regression of predicted (biological) on observed (chronological) age is defined as the age acceleration (or deceleration in the case of negative values) according to the physical fitness revealed by the set of physical tests administered. In this context, fitAA may be used as a quantitative measure of the difference between biological and chronological age, allowing the identification of individuals experience accelerated (or decelerated) aging.

When we measured the ability of this newly introduced measure to predict health status in terms of its association with risk scores for CVD and mortality and compared it with the conventional definition of fitness based on the 6MWT, we found that fitAA, unlike 6MWT, was significantly associated with major health risk scores calculated according to established formulae (D’Agostino et al., 2008; Goff et al., 2013; Budoff et al., 2018; Levine et al., 2018; Roffman et al., 2016): the higher fitAA, the higher the short term risk of cardiovascular events and death.

To improve the interpretability of our results we categorized individuals into three groups according to their fitAA value: individuals with fitAA lower than −2.5 years (labelled as “FIT”) showed the highest fitness and lowest risk scores, suggesting that they experience slower and healthier ageing. Conversely, individuals with fitAA higher than +2.5 years showed the lowest fitness and highest risk scores, suggesting accelerated and less healthy ageing. Scores between these two cut-offs characterise individuals whose biological age is close to their chronological age and with intermediate risk scores.

Such estimates can be interpreted as the increase in the risk score of “NORMAL” and “UNFIT” individuals compared to the “FIT” group used as the reference. Accordingly, we estimated that an “UNFIT” individual has, on average, around 5% higher probability of dying or experiencing a CVD event within the next 10 years than a “FIT” individual. This categorization allowed us to compare our multi-level measure with that based on the conventional 6MWT. This study’s results highlight that a composite measure of fitness status outperforms 6MWT in estimating the state of health of an individual.

This work has limitations. First, we collected data from a mixed cohort of middle-aged and older adults. Further, the available sample size does not allow us to generalise these results to the whole population over 50 years and does not allow setting-up cut-offs for the definition of accelerated, normal, or decelerated aging. However, we provided proof of the advantages of using multiple fitness tests to assess the health status of the elderly. The weights defined in this study through the elastic net regression model must be calibrated and validated in larger population studies before they can be used in clinical practice.

Further, additional studies are needed to assess fitness status separately in women and men, as the measures to evaluate it in the elderly and the transition from adult age to elderly may behave differently in the two sexes, even though our data consistently ranked the TUG as the major contributor to biological age in both men and women.

## Conclusion

Our results suggest that a proper evaluation of fitness status should favor a set of physical motor tests that include the TUG, which reflects the essential components of mobility, and handgrip strength, which is a valid indicator of overall strength. These two simple tests proved the best predictors of fitness age and could represent robust and feasible tools to monitor the ageing process according to the fitness level displayed. In conjunction with this finding, the newly introduced measure—fitAA—that quantifies the difference between biological and chronological age—can help to identify individuals at high risk for non-communicable diseases in the short period, with important advantages for public health and screening policies. Although we examined well-validated measures of risk to assess the individual state of health, further investigation using a longitudinal design is needed to assess risk measures associated with accelerated fitAA more precisely and to validate our findings.

## Data Availability

The raw data supporting the conclusion of this article will be made available by the authors, without undue reservation.
