# Addendum: A novel estimate of biological aging by multiple fitness tests is associated with risk scores for age-related diseases

**DOI:** 10.3389/fphys.2025.1738830

**Published:** 2025-12-19

**Authors:** A. Manca, G. Fiorito, M. Morrone, A. Boi, B. Mercante, G. Martinez, L. Ventura, A. P. Delitala, A. Cano, M. G. Catte, G. Solinas, F. Melis, F. Ginatempo, F. Deriu

**Affiliations:** 1 Department of Biomedical Sciences, University of Sassari, Sassari, Italy; 2 Department of Medicine, Surgery and Pharmacy, University of Sassari, Sassari, Italy; 3 Unit of Endocrinology, Nutritional and Metabolic Disorders, AOU Sassari, Sassari, Italy

**Keywords:** biological age-chronological age, aging, cardiovascular diseases, 6-min walking test, timed “up and go” test, ten meter walk test, handgrip test

The formula by which Fitness Age is calculated was not reported in the section **Results**, *Fitness age definition and components,* below table 2:

“ $FitAge=66.48-\frac{2.23*\;\left(TUG_{time}-6.69\right)}{1.38}-\frac{1.98*\;\left(Handgrip_{normalized}-0.37\right)}{0.11}-$ $\frac{1.11*\left({6MWT}_{distance}-502.38\right)}{74.94}+0.95\;*\;\frac{\left({Fast10MWT}_{time}-4.75\right)}{0.93}+0.23*\frac{\left({4SST}_{time}-3.67\right)}{0.81}-0.19$ $*\frac{\left({StrongerQuadStrength}_{normalized}-0.56\right)}{0.13}$

Where means (µ) and standard deviations (σ) were computed on this dataset:

TUG_time_ (s): µ = 6.69; σ = 1.38

Handgrip_normalized_ (by bodyweight in kg): µ = 0.37; σ = 0.11

6MWT_distance_ (m): µ = 502.38; σ = 74.94

fast10MWT_time_ (s): µ = 4.75; σ = 0.93

4SST_time_ (s): µ = 3.67; σ = 0.81

StrongerQuadStrength_normalized_ (by bodyweight in kg): µ = 0.56; σ = 0.13” 

